# Supplementary material for: Antineoplastic Nature of WWOX in Glioblastoma Is Mainly a Consequence of Reduced Cell Viability and Invasion
Source: Biology (Basel). 2023 Mar 17;12(3):465. doi: 10.3390/biology12030465 (PMC10045224; doi:10.3390/biology12030465)
Supplement: Supplementary file 1 [file biology-12-00465-s001.zip › Table_S1.pdf]

Table S1. Data acquired from cBioPortal and CCLE on the mutational status, CNAs, and mRNA expression of genes identified throughout the discussion of results.

	T98G			U87MG			U251MG			DBTRG-05MG		
	CNA#	Mut.*	mRNA expression	CNA	Mut.	mRNA expression	CNA	Mut.	mRNA expression	CNA	Mut.	mRNA expression
**AKT1**	no alt.†		43.62067	no alt.		39.27408	no alt.		32.03077	no alt.		47.24887
**BRAF**	no alt.		3.48314	no alt.		4.18347	no alt.		5.05805	no alt.	V600E	3.28902
**CDH1**	no alt.		0.07831	no alt.		0.08822	no alt.		0.15062	no alt.		0.2802
**ELF5**	no alt.		0.01236	no alt.		0.4491	no alt.		0.01266	no alt.		0.05916
**IDH1**	no alt.		44.8614	no alt.		51.77509	no alt.		40.65739	no alt.		35.94795
**IDH3G**	no alt.		26.27039	no alt.		24.18385	no alt.		25.21216	no alt.	R109W	28.79707
**RUNX2**	no alt.		13.01955	no alt.		4.66042	no alt.		8.68072	no alt.		3.81298
**SMAD3**	no alt.		38.82957	no alt.		21.21619	no alt.		25.25521	no alt.		18.77967
**SNAI1**	no alt.		8.11037	no alt.		0.60624	no alt.		0.63004	no alt.		1.75119
**TNK2 (ACK1)**	no alt.		6.85204	no alt.		6.51467	no alt.		9.53257	no alt.		4.93732
**TP53**	no alt.	M237I	48.94476	no alt.		7.00334	no alt.	R273H	38.63569	no alt.		14.73492

# – copy number alteration; * – mutation; † – no alteration.
